# Blood lactate accumulation during maximal cycling sprints and its relationship to sprint performance characteristics

**DOI:** 10.1007/s00421-025-05755-9

**Published:** 2025-03-20

**Authors:** Ralf Haase, Anna Katharina Dunst, Nico Nitzsche

**Affiliations:** 1 Department of Sports Medicine and Exercise Therapy, Institute of Human Movement Science and Health, Chemnitz University of Technology, Thüringer Weg 11, 09126 Chemnitz, Germany; 2 Department of Endurance Sports, Institute for Applied Training Science Leipzig, Marschnerstraße 29, 04109 Leipzig, Germany

**Keywords:** Anaerobic performance, Anaerobic diagnostics, Exercise testing, Glycolysis

## Abstract

**Purpose:**

Blood lactate accumulation (ΔBLC) during maximal short-term exercise is a crucial indicator of peak glycolytic activation in anaerobic performance assessment. However, the relationship between ΔBLC and sprint performance remains inconsistent, potentially due to variations in testing protocols and the use of absolute rather than relative performance metrics. This study investigated the relationship between ΔBLC and cycling sprint performance, hypothesizing normalization to body weight is essential for accurate metabolic performance evaluation.

**Methods:**

Twenty-two trained male athletes performed a 10-s maximal isokinetic cycling sprint on an ergometer. Power output and cadence were continuously recorded to calculate peak power (*P*_peak_), time to peak power (*t*_*P*peak_), mean power (*P*_mean_), and power increase during the lactic phase (maxΔ*P*, Δ*P*). Capillary blood samples were collected pre-exercise and up to 12 min post-exercise to determine pre-exercise (BLC_pre_) and maximal post-exercise blood lactate concentration (BLC_max_). ΔBLC was calculated as BLC_max_−BLC_pre_. Statistical analysis included Pearson correlations and stepwise multiple regression.

**Results:**

ΔBLC exhibited significant correlations with body-weight-normalized maxΔ*P* (*r* = 0.78, *p* < 0.001), *P*_mean_ (*r* = 0.70, *p* < 0.001), and *P*_peak_ (*r* = 0.65, *p* < 0.01). In contrast, no significant correlations were observed with absolute metrics (*p* > 0.05). Stepwise regression analysis identified adjusted maxΔ*P* and *P*_mean_ as the strongest predictors of ΔBLC (adjusted *R*^2^ = 0.648, *p* < 0.001).

**Conclusion:**

Relative, body-weight-adjusted metrics, particularly maxΔ*P* and *P*_mean_, are strongly associated with ΔBLC. The use of these relative metrics may enhance the precision of anaerobic performance assessment, facilitate more effective training monitoring, and improve talent identification processes in sports requiring high-intensity efforts.

## Introduction

Understanding the metabolic pathways that supply adenosine triphosphate (ATP) during maximal exercise is essential for optimizing training intensity, load, and recovery (Gastin 2001). Indirect calorimetry, which measures oxygen uptake and lactate accumulation before, during, and after exercise, effectively assesses the contributions of anaerobic alactic, lactic, and aerobic energy pathways (Di Prampero 1981; Kaufmann et al. 2022; Mader 1994).

During maximal sprinting, anaerobic alactic metabolism (phosphagen system) primarily fuels the initial phase, where phosphocreatine (PCr) dephosphorylation meets energy demands. PCr degradation reaches its maximum within approximately 1.3 s and decreases thereafter, while the rate of glycolysis increases until 5 s of onset (Gastin 2001; Maughan et al. 1997). Furthermore, during supramaximal sprints, it has been shown that PCr stores are depleted after 5–7 s (Hirvonen et al. 1987). Approximately half of the PCr stores can be depleted without significantly affecting ATP concentration or muscle performance (Chung et al. 1998; Di Prampero 1981; Dunst et al. 2023a, b). Following this initial phase, ATP is predominantly resynthesized via anaerobic glycolysis from approximately 3–12 s of the maximal effort (Baker et al. 2010; Dunst et al. 2023a, b; Heck et al. 2003; Jones et al. 1985). During this period, lactate is rapidly produced from pyruvate in the muscle fibers and is extruded with hydrogen ions (H^+^) via monocarboxylate transporters, leading to blood lactate accumulation (Juel and Halestrap 1999). The rising H^+^ concentration inhibits key glycolytic enzymes, reducing glycolytic energy flux and necessitating a transition to oxidative phosphorylation to meet energy demands. However, the lower energy flux associated with oxidative metabolism results in a subsequent decrease in power output (Heck et al. 2003; Mader 2003). Despite the reduction in glycolytic flux, anaerobic glycolysis continues to contribute significantly to ATP resynthesis during sprints up to 400 m or maximal efforts lasting up to 60 s (Duffield et al. 2004, 2005; Heck et al. 2003; Spencer and Gastin 2001). Therefore, short maximal efforts of 3–12 s lead to rapid blood lactate accumulation (ΔBLC) due to high to maximal glycolytic activity, which facilitates rapid ATP resynthesis during intense exercise (Baker et al. 2010; Heck et al. 2003).

The maximal rate of blood lactate accumulation, referred to as *v*La_max_, serves as an indicator of the maximal glycolytic flux specific to the exercise performed (Mader 2003). However, after approximately 3 s, the lower rate of glycolytic ATP production (1.5–3 mmol kg^−1^ s^−1^ in wet muscle) contributes to a decline in muscle performance and power output (Crowther et al. 2002; Dunst et al. 2023a, b; Heck et al. 2003; Mader 2003). The maximal glycolytic rate achieved by the muscles depends largely on the availability of glycolytic enzymes, particularly phosphofructokinase (Heck et al. 2003).

Elite sprinters typically exhibit higher glycolytic rates and rapid blood lactate accumulation, attributed to a greater proportion of fast-twitch (type II) muscle fibers, which have a higher glycolytic capacity than type I fibers (Esbjörnsson-Liljedahl et al. 1999; Essén-Gustavsson and Henriksson 1984). Recent research showed that professional track cycling sprinters display *v*La_max_ values exceeding 1.0 mmol L^−1^ s^−1^ with maximal cadences above 300 rpm (Dunst et al. 2021; Dunst et al. 2023a, b), indicating a significant proportion of fast-twitch fibers (Hansen et al. 2002; Hautier et al. 1996). Fast-twitch fibers, known for their high contraction velocity and force production at greater movement speeds, are closely linked to enhanced sprint performance (Bottinelli et al. 1996). This suggests a relationship between glycolytic activity, blood lactate accumulation, and sprint performance in short maximal sprints.

Despite, the relationship between blood lactate concentration and exercise performance remains controversial, with conflicting results across studies (Takei et al. 2018). Some research have reported moderate to high correlations between blood lactate measures and sprint performance in both running and cycling, with variations based on sprint duration and training status. Strong correlations have been observed in maximal sprints up to 30 s, particularly in untrained individuals, suggesting that factors such as training status, and individual physiological adaptations may influence the strength of this association (Burke et al. 1981; Fujitsuka et al. 1982; Gupta et al. 2021; Korhonen et al. 2005; Lacour et al. 1990; Langley et al. 2024; Ohkuwa et al. 1984; Quittmann et al. 2020, 2021). However, other research did not find significant correlations (Ohkuwa et al. 1984; Takei et al. 2018).

Contradictory findings may arise from several factors, including variations in exercise type, test duration, and measurement methods. While both maximal post-exercise blood lactate concentration (BLC_peak_) and ΔBLC have been used as indicators of glycolytic activity, BLC_peak_ is strongly influenced by pre-exercise blood lactate levels (BLC_pre_). In contrast, ΔBLC mathematically eliminates the influence of BLC_pre_ but may be affected if BLC_pre_ is significantly elevated above resting levels, potentially impairing glycolytic activity (Pohl et al. 2024). The relationship between pre-exercise lactate levels, glycolytic activity, and performance measures highlights the importance of carefully controlling testing conditions and interpreting blood lactate data in exercise physiology research.

Test duration also significantly influences the interpretation of blood lactate data and its relationship to performance (Langley et al. 2024; Mavroudi et al. 2023). While anaerobic metabolism contributes substantially to energy production during 60-s maximal exercises, the direct impact of maximal glycolysis on sprint performance is most pronounced within the first 12 s, before aerobic metabolism becomes increasingly dominant (Dunst et al. 2023a, b; Spencer and Gastin 2001). This time-dependent shift in energy system contribution underscores the importance of carefully selecting test durations that align with the specific metabolic processes under investigation, particularly when examining the relationship between blood lactate accumulation and short-duration sprint performance. Recent research identified a 10-s maximal effort as the optimal duration for assessing *v*La_max_ (Langley et al. 2024).

A further critical limitation of previous studies has been the insufficient consideration of body composition when analyzing performance metrics. Research has demonstrated strong correlations between mechanical peak power output (*P*_peak_) and leg muscle mass in cycling

(Kordi et al. 2020), as well as between mean power output (*P*_mean_) and *P*_peak_ in maximal cycling sprints lasting up to 60 s (Dunst and Grüneberger 2021; Dunst et al. 2021). These inherent relationships between body composition, muscle mass, and power output can potentially mask the true association between mechanical power output and metabolic performance. This assumption is further supported by recent findings indicating that the combination of fat-free mass and glycolytic energy contribution strongly predicts performance in a 15-s maximal cycling sprint (Meixner et al. 2024). Failing to account for these factors may lead to misinterpretations of the relationship between blood lactate measures and sprint performance, particularly when comparing athletes with different body compositions or across varying levels of training status. Therefore, adjusting performance metrics for body weight or lean muscle mass as indicators of maximal neuromuscular performance is crucial for isolating and accurately assessing the metabolic aspects of sprint performance.

To address these gaps, this study aims to investigate the relationship between ΔBLC, as a surrogate for glycolytic performance, and relative performance metrics in a maximal 10-s cycling sprint in healthy active males, while controlling for pre-exercise blood lactate concentration. We hypothesize that, under controlled test conditions, significant correlations between ΔBLC and performance metrics relative to body weight will be observable in samples that are either heterogeneous or homogeneous in *P*_peak_. Given the assumption that higher glycolytic activity correlates with higher performance, participants that are more powerful are expected to exhibit greater ΔBLC values. However, performance metrics without normalization to body weight may show weaker associations in these individuals due to the greater impact of maximal strength and power on performance data.

This research will contribute to a better understanding of the relationship between blood lactate accumulation and sprint performance, potentially improving the assessment of anaerobic power in athletes.

## Methods

### Participants

Twenty-two trained male athletes (age: 31.8 ± 9.8 years, height: 1.79 ± 0.1 m, body mass: 77.6 ± 12.8 kg, body mass index: 24.1 ± 3.3 kg m^−2^) participated in the study. The participants reported a mean training volume of 8.0 ± 5.4 h per week of training across different sport disciplines including cycling, running, resistance training, handball, and fitness sport. The participants were familiar with the sprint test from their involvement in previous studies. Participants were instructed to avoid strenuous and/or high-volume physical activities for at least 24 h prior to the experimental session. They were further instructed to maintain their dietary habits and to refrain from consuming alcohol. All participants provided written informed consent after being fully informed of the tests and associated risks. The study was appraised by the local ethics committee (#101594111) and was conducted according to the Declaration of Helsinki (latest version).

### Exercise protocol

Each participant completed a 10-s maximal isokinetic cycling sprint at a pedaling frequency of 130 revolutions per minute (rpm) on a cycle ergometer (Excalibur Sport, Lode B. V., Groningen, Netherland). Prior to the sprint test, the participants performed a 10-min warm-up, cycling at a workload corresponding to 0.5 Watt per kilogram bodyweight while maintaining a pedaling frequency of 60–80 rpm. The warm-up was followed by a short break to collect one capillary blood sample to determine the pre-exercise BLC (BLC_pre_) and to switch the ergometer to isokinetic mode. During the sprint test, the participants were instructed to remain seated and were verbally encouraged.

### Measurements and data processing

**Fig. 1:**
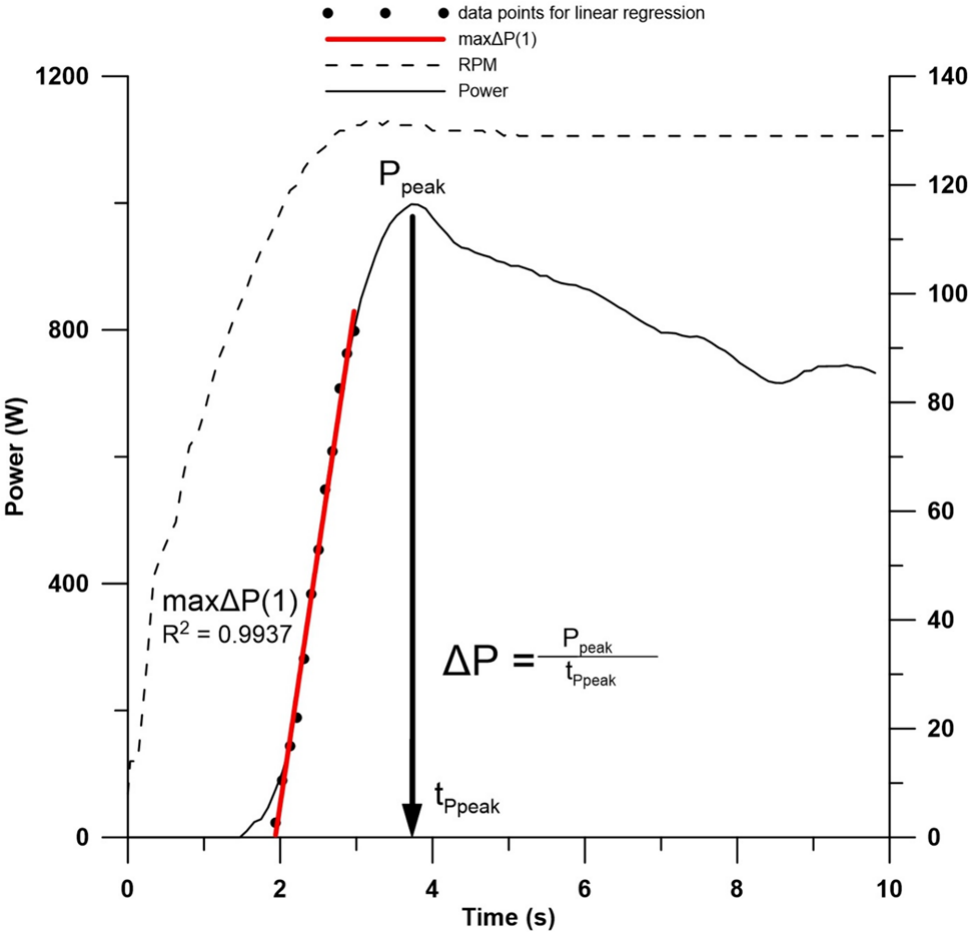
Representative power output curve from a 10-s maximal isokinetic cycling sprint, illustrating key performance metrics (*P*_*peak*_ peak power output, *P*_*mean*_ mean power output, *ΔP* power increase calculated by *P*_peak_/*t*_*P*peak_, *maxΔP* strongest power increase over 1 s, *rpm* revolutions per minute, *t*_*P*peak_ time to reach *P*_peak_)

Power and pedaling frequency were continuously measured during each sprint at a sampling rate of 10 Hz. Peak power output (*P*_peak_) and its corresponding time-point (*t*_*P*peak_) were determined. The mean power output (*P*_mean_) for the 10-s sprint was calculated from recorded values. The ergometer used an eddy current brake to adjust workload and maintain constant pedaling frequency. After 2–3 s, when the targeted pedaling frequency was nearly reached, the eddy current brake increased the workload according to the athlete’s force input. The initial increase of the power until *P*_peak_ is reached was calculated by dividing *P*_peak_ by *t*_*P*peak_ and termed ΔP. The steepest increase in power output over a one-second period, determined by linear regression, was defined as maxΔ*P* (Fig. 1). All power metrics (*P*_peak_, *P*_mean_, Δ*P*, maxΔ*P*) were also expressed relative to body weight (rel*P*_peak_, rel*P*_mean_, relΔ*P*, rel.maxΔ*P*). All power outputs were checked for plausibility, to verify for an all-out effort. If any deviations were observed, for example reaching *t*_*P*peak_ very late or reaching multiple peaks in power, the sprint test were repeated.

Capillary blood samples (20 μl) were collected from the hyperemic earlobe (Finalgon^®^) before and after the test. The first sample was taken immediately before the 10-s sprint, followed by samples at the end of the sprint and at 1, 3, 5, 7, 9, and 12 min post-sprint. Blood lactate concentration was determined using the enzymatic amperometric method (Biosen C-Line, EKF-diagnostics GmbH, Barleben, Germany).

ΔBLC was calculated as the difference of BLC_max_ and BLC_pre_. To determine BLC_max_ and its corresponding time point (TBLC_max_), a bi-exponential function was used:

1 $${\text{BLC(t) = }}\frac{{{\text{A }} \cdot {\text{k}}_{{1}} }}{{{\text{k}}_{{2}} - {\text{ k}}_{{1}} }}{ } \cdot {\text{ (e}}^{{ - {\text{k}}_{{1}} { } \cdot {\text{ t}}}} - {\text{e}}^{{ - {\text{k}}_{{2}} { } \cdot {\text{ t}}}} {\text{) + BLC}}_{{{\text{pre}}}}$$

where A represents the extravascular increase in lactate concentration, k_1_ is the invasion constant of lactate into the blood compartment, and k_2_ is the elimination constant of lactate out of the blood compartment (Beneke et al. 2005). Model parameters A, k_1_ and k_2_ were determined by non-linear regression analysis. The *v*La_max_ were determined according to Eq. 2 with *T*_load_ being the test time of 10 s and *t*_alac_ the fictional alactic time. For *t*_alac_ a fixed time of 3 s were applied.

2 $${\text{v}}{\text{La}}_{\text{max}}\text{=} \frac{{\text{BLC}}_{\text{max}} - \, {\text{BLC}}_{\text{pre}}}{{\text{T}}_{\text{load}} - \, {\text{t}}_{\text{alac}}}$$

### Statistics

Data were presented as mean, standard deviation (SD) and coefficient of variation (CV). Normality of distribution was evaluated using the Shapiro–Wilk test. Depending on data normality, either Pearson’s r or Spearman’s rho (*ρ*) were calculated to examine correlations between performance metrics and ΔBLC. The correlation coefficients were interpreted as follows:* r* = 0.10 small, *r* = 0.30 medium, *r* = 0.50 large (Cohen 1988). To distinguish between participants with high and low relative power output, the sample was divided based on the median of relative peak power (rel*P*_peak_). The group comparisons were conducted using independent *t*-tests and interpreted using Cohen’s *d* (*d* = 0.20 small, *d* = 0.50 medium, *d* = 0.80 large; Cohen 1988). Variables most strongly correlated with ΔBLC were selected for a backward stepwise regression analysis, with exclusion criteria set at *p* > 0.1. These variables were correlated with each other, to check if there is a redundancy of information. For the regression analysis, *z*-scores were calculated for ΔBLC and the included parameters using the sample mean and SD (Sedlmeier and Renkewitz 2018). The assumptions of normal distribution of the error, independence of the error, homoscedasticity and multicollinearity were visually checked or with the respective statistical test (variance inflation factor). Goodness of fit were evaluated using *R*^2^, adjusted *R*^2^ (small 0.02, medium 0.13, large 0.26) and the standard error of the estimate (SEE) (Cohen 1988). To validate the backward stepwise regression results, nested models with fewer parameters were compared using the likelihood ratio test (Wolf and Best 2010). The significance level was set at 0.05.

Statistical analyses were performed using jamovi (Version 2.3.28.0) and R (version 4.3.2, package lmtest, version 0.9–40). Data processing was performed in Microsoft Office Excel 2019 (Microsoft Corporation, Redmond, WA, USA). The figures were created using Grapher 12 (Golden Software, LLC, Golden, CO, USA).

## Results

The exercise protocol effectively maintained pre-exercise blood lactate concentrations below 2 mmol/L for all participants, ensuring standardized initial metabolic conditions (Pohl et al. 2024). All athletes reached the target pedaling frequency before the end of the highest rate of power increase, which ended after about 3–4 s, indicating significant glycolytic involvement during the acceleration phase (Dunst et al. 2023a, b). Figure 1 shows a representative power output curve from a 10-s sprint, illustrating key performance metrics including *P*_peak_, *P*_mean_, *t*_*P*peak_, and maxΔ*P* during the lactic phase (Δ*P*, maxΔ*P*).

The results of the 10-s maximal isokinetic cycling sprint tests are presented in Table 1.

**Table 1:** Power output and blood lactate concentration metrics for the 10-s maximal isokinetic cycling sprint

	Overall (*n* = 22)		Low rel*P*_peak_ (< 14.09 W kg^−1^, *n* = 11)		High rel*P*_peak_ (> 14.09 W kg^−1^, *n* = 11)		*p*	*d*
Parameters	Mean ± SD	CV (%)	Mean ± SD	CV (%)	Mean ± SD	CV (%)		
*P*_Peak_ (W)	1090.36 ± 175.18	16.1	1062.64 ± 202.32	19.0	1118.09 ± 147.71	13.2	0.471	0.31
rel*P*_Peak_ (W kg^−1^)	14.09 ± 1.02	7.3	13.35 ± 0.62	4.6	14.83 ± 0.78	5.5	< 0.001**	2.12
*P*_mean_ (W)	658.01 ± 144.30	21.9	657.62 ± 164.86	25.1	658.40 ± 128.63	19.5	0.990	0.01
rel*P*_mean_ (W kg^−1^)	8.42 ± 0.76	9.0	8.18 ± 0.72	8.7	8.67 ± 0.76	8.7	0.138	0.66
Δ*P*	264.75 ± 78.40	29.6	260.63 ± 81.09	31.1	268.86 ± 79.34	29.5	0.812	0.10
relΔ*P*	3.36 ± 0.55	16.3	3.22 ± 0.49	15.3	3.50 ± 0.59	16.8	0.231	0.53
maxΔ*P* (W s^−1^)	864.02 ± 157.24	18.2	831.99 ± 166.62	20.0	896.037 ± 48.01	16.5	0.352	0.41
rel.maxΔ*P* (W kg^−1^ s^−1^)	11.15 ± 1.09	9.7	10.45 ± 0.68	6.5	11.85 ± 0.96	8.2	< 0.001**	1.69
*t*_*P*peak_ (s)	4.28 ± 0.62	14.6	4.22 ± 0.57	13.6	4.33 ± 0.70	16.0	0.685	0.18
BLC_pre_ (mmol L^−1^)	0.88 ± 0.25	28.5	0.79 ± 0.18	22.2	0.98 ± 0.29	29.6	0.087	0.77
BLC_max_ (mmol L^−1^)	6.39 ± 0.83	12.9	6.02 ± 0.73	12.1	6.76 ± 0.78	11.5	0.032*	0.98
ΔBLC (mmol L^−1^)	5.51 ± 0.72	13.1	5.23 ± 0.64	12.3	5.79 ± 0.71	12.3	0.068	0.82
*v*La_max_ (mmol L^−1^ s^−1^)	0.79 ± 0.10	13.1	0.75 ± 0.09	12.3	0.83 ± 0.10	12.3	0.067	0.83
TBLC_max_ (min)	3.36 ± 0.75	22.3	3.22 ± 0.72	22.3	3.51 ± 0.79	22.5	0.377	0.39

Data are presented as mean ± standard deviation (SD) with coefficient of variation (CV) and total range for the overall sample and subdivided by the median relP_peak_ (14.09 W kg^−1^)

*ΔBLC* blood lactate accumulation (BLC_max_–BLC_pre_), *BLC*_*pre*_ pre-exercise blood lactate concentration, *BLC*_*max*_ maximal capillary blood lactate concentration post-exercise, *CV* coefficient of variation, *P*_peak_ peak power output, *P*_mean_ mean power output, *ΔP* power increase calculated by *P*_peak_/*t*_*P*peak_, *maxΔP* strongest power increase over 1 s, *rel* power parameter relative to the bodyweight, *t*_*Ppeak*_ time to reach peak power output, *TBLC*_*max*_ time of occurrence of BLC_max_, *vLa*_*max*_ maximal rate of blood lactate accumulation

**Fig. 2:**
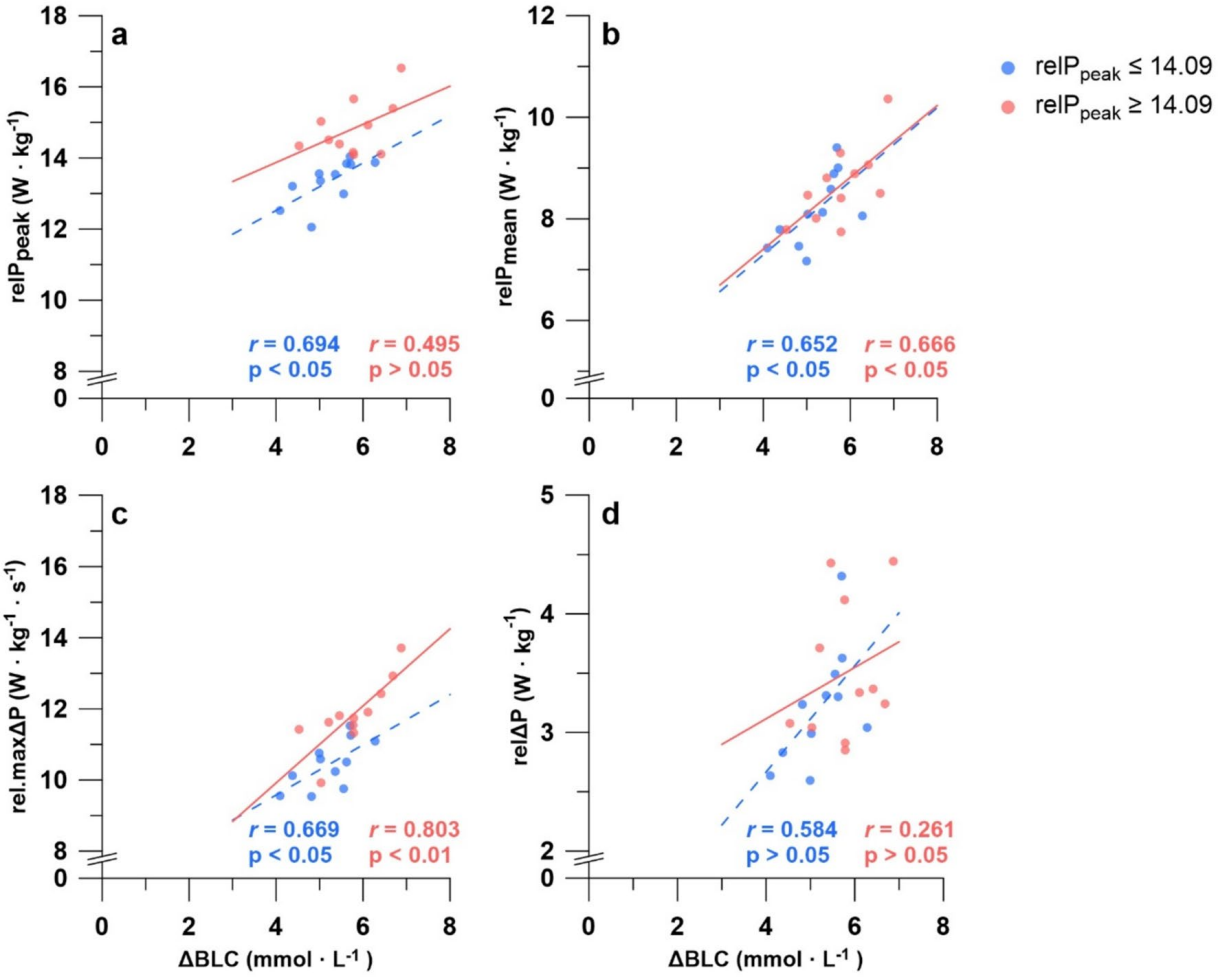
Scatterplots of the blood accumulation (ΔBLC = BLC_max_–BLC_pre_) and different parameters of the performance during the 10 s maximal isokinetic cycling sprint. **a** Correlation between ΔBLC and rel*P*_peak_, **b** Correlation between ΔBLC and rel*P*_mean_, **c** Correlation between ΔBLC and rel.maxΔ*P*, **d** Correlation between ΔBLC and relΔ*P* (*relP*_peak_ relative peak power output, *relP*_mean_ relative mean power output, *rel.maxΔP* strongest power increase over 1 s relative to the bodyweight, *relΔP* power increase calculated by *P*_peak_/*t*_*P*peak_ relative to the bodyweight)

A very strong significant correlation was found between *P*_peak_ and the body mass (*r* = 0.907, *p* < 0.001). No significant correlations were observed between *P*_peak_, *P*_mean_, Δ*P*, maxΔ*P* in absolute values and ΔBLC. However, when adjusted for body mass, significant correlations emerged between ΔBLC and the different power parameters for the overall and subdivided sample (Table 2). Since a fixed *t*_alac_ of 3 s was used in the calculation of *v*La_max_, the correlations for *v*La_max_ are identical to those between ΔBLC and the power parameters (Table 2). Small differences were observed between the magnitude of the correlations according to the group. The results are shown in Fig. 2. Significantly higher BLC_peak_ was observed in the group with higher rel*P*_peak_ (*p* < 0.05). This did not translate to ΔBLC, which showed a higher increase of BLC in the high rel*P*_peak_ group, but not statistically significant (*p* = 0.068).

**Table 2:** Correlations between ΔBLC and power parameters overall and subdivided by the median rel*P*_peak_ (14.09 W kg^−1^)

Parameters	ΔBLC Overall (*n* = 22)	ΔBLC low rel*P*_peak_ (< 14.09 W kg^−1^, *n* = 11)	ΔBLC high rel*P*_peak_ (> 14.09 W kg^−1^, *n* = 11)
*P*_Peak_ (W)	0.259	0.296	0.126
rel*P*_Peak_ (W kg^−1^)	0.649**	0.694*	0.495
*P*_mean_ (W)	0.269	0.341	0.245
rel*P*_mean_ (W kg^−1^)	0.702***	0.652*	0.666*
Δ*P*	0.236	0.383	0.099
relΔ*P*	0.459*	0.584	0.261
maxΔ*P* (W s^−1^)	0.379	0.336	0.329
rel.maxΔ*P* (W kg^−1^ s^−1^)	0.775***	0.669*	0.803**
*t*_*P*peak_ (s)	− 0.191	− 0.425	− 0.119

*ΔBLC* blood lactate accumulation (BLC_max_–BLC_pre_), *BLC*_*pre*_ pre-exercise blood lactate concentration, *BLC*_*max*_ maximal capillary blood lactate concentration post-exercise, *CV* coefficient of variation, *P*_peak_ peak power output, *P*_mean_ mean power output, *maxΔP* strongest power increase over 1 s, *ΔP* power increase calculated by *P*_peak_/*t*_*P*peak_, *rel* power parameter relative to the bodyweight, *t*_*P*peak_ time to reach peak power output, *p < 0.05; **p < 0.01; ***p < 0.001

Therefore, backward stepwise regression analysis focused on relative power metrics: rel*P*_peak_, rel*P*_mean_, relΔ*P*, and rel.maxΔ*P*. Correlation analysis among the parameters revealed strong associations between rel.maxΔ*P* and rel*P*_peak_ (*r* = 0.82), as well as between rel*P*_mean_ and relΔ*P* (*r* = 0.80). Moderate correlations were observed between rel.maxΔ*P* and rel*P*_mean_ (*r* = 0.62), rel.maxΔ*P* and relΔ*P* (*r* = 0.51), and rel*P*_mean_ and rel*P*_peak_ (*r* = 0.54). These correlations indicate potential multicollinearity among the predictors, which was addressed through the stepwise regression process. The final predictive model for ΔBLC incorporated rel.maxΔP and rel*P*_mean_, yielding a statistically significant outcome (adjusted *R*^2^ = 0.648, F(2, 19) = 20.297, *p* < 0.001). The model demonstrated a standard error of estimate (SEE) of 0.594 (*z*-score) and 0.428 mmol L^−1^ (unstandardized) (Table 3). The likelihood ratio test revealed no significant difference between the full model (including all four power parameters) and the reduced model after the backwards regression *Χ*^2^ (2) = 2.533, *p* > 0.05. A further reduction of the model resulted in significant differences between the models with *Χ*^2^ (1) = 10.235, *p* < 0.01 when rel.maxΔ*P* was removed and *Χ*^2^ (1) = 4.962, *p* < 0.05 when rel*P*_mean_ was removed, reducing the adjusted *R*^2^ in these models (0.49 and 0.60, respectively).

**Table 3:** Results of the backwards stepwise regression analysis to predict ΔBLC

Parameters	Coefficients (*z*-score)	Coefficients (unstandardized)	SE (*z*-score)	95% CI (*z*-score)	T (*z*-score)	*p*-value (*z*-score)
Intercept	0.00	− 1.465	0.127	− 0.26 –0.26	0.000	> 0.05
rel.maxΔ*P* (W kg^−1^ s^-1^)	0.552	0.367	0.165	0.21 –0.90	3.355	< 0.01
rel*P*_mean_ (W kg^−1^)	0.361	0.342	0.165	0.02 –0.71	2.193	< 0.05

*CI* confidence interval, *ΔBLC* blood lactate accumulation (BLC_max_–BLC_pre_), *BLC*_*pre*_ pre-exercise blood lactate concentration, *BLC*_*max*_ maximal capillary blood lactate concentration post-exercise, *P*_*mean*_ mean power output, *maxΔP* strongest power increase over 1 s, *rel* power parameter relative to the bodyweight, *SE* standard error

## Discussion

This study aimed to address existing research gaps by investigating the relationship between ΔBLC, as a surrogate for glycolytic performance and relative performance metrics. For this purpose, a maximal 10-s cycling sprint was conducted with healthy active males, while controlling for pre-exercise blood lactate concentration. As hypothesized, statistically significant correlations were found between ΔBLC and sprint performance only when expressed relative to body weight in samples that are either heterogeneous or homogeneous in *P*_peak_, whereas no significant relationships were found for absolute values.

A strong correlation between mean power output and ΔBLC was observed when *P*_mean_ was normalized to body weight (Table 2). This adjustment is physiologically relevant, as body weight normalization accounts for the influence of *P*_peak_, which strongly correlated with *P*_mean_. Previous studies have provided a framework for understanding sprint performance, modeling it with the equation *P*(t) = *A*‧e^−t/τ^ + c, where *A* represents the amplitude, *τ* is the time constant of power decline and c denotes the asymptotic limit of anaerobic efforts (Dunst and Grüneberger 2021; Dunst et al. 2021). Sensitivity analyses demonstrated that mean power output, the integral of *P*(t) over the test duration, is predominantly determined by *P*_Peak_ = A + c, which reflects neuromuscular performance (Dunst and Grüneberger 2021; Dunst et al. 2021). The time constant *τ*, however, is associated with cadence and metabolic performance (Dunst et al. 2021). This distinction underscores the need to control for neuromuscular contributions when aiming to isolate metabolic performance. By normalizing power output to body weight, which was strongly correlated with *P*_peak_, our study effectively mitigated the confounding influence of neuromuscular performance, providing a clearer assessment of the relationship between ΔBLC and *P*_mean_.

Further support for the importance of body composition in sprint performance comes from recent findings by Meixner et al. (2024). They demonstrated that work generated during an all-out 15-s isokinetic cycling sprint is strongly influenced by fat-free mass. Moreover, their results showed that the combination of fat-free mass and glycolytic energy contribution (estimated via ΔBLC and fat-free mass) provides a robust prediction of total work output. These findings align with our conclusion that *P*_mean_ is significantly associated with ΔBLC only when adjusted for body mass.

Notably, this study is the first to demonstrate a significant relationship between ΔBLC and the highest power increase during a maximal isokinetic cycling sprint when normalized by body weight (Fig. 2, Table 2). Similarly, *P*_peak_ was significantly correlated with ΔBLC only when adjusted for body weight (Fig. 2, Table 2). Since both *P*_peak_ and maxΔ*P* were achieved during the lactic phase of the effort (Dunst et al. 2023a, b), they are influenced by both neuromuscular performance and maximal glycolytic flux. By normalizing these metrics relative to body composition, the confounding effect of neuromuscular performance may be mitigated, allowing for a clearer assessment of metabolic performance. These findings suggest that body composition-adjusted metrics provide a more accurate reflection of glycolytic performance in short maximal efforts.

A multiple regression analysis comparing the predictive potential of power metrics correlated with ΔBLC revealed that rel.maxΔ*P* and rel*P*_mean_ were strongly associated with ΔBLC, explaining approximately 64.8% of the variance in blood lactate accumulation during maximal short-term cycling exercise (Fig. 2, Table 3). The differing predictive potential of rel.maxΔ*P* and rel*P*_mean_ likely reflect the distinct physiological processes these metrics capture. When adjusted for body weight, *P*_mean_ provides a comprehensive measure of total energy supply during maximal efforts, incorporating contributions from alactic anaerobic, lactic anaerobic, and aerobic energy systems (albeit with only small aerobic involvement under these conditions; Langley et al. 2024; Yang et al. 2023). While this integrated measure reflects overall energetic capacity, it may mask the contributions of individual energy systems. In contrast, rel.maxΔ*P* likely better reflects maximal glycolytic flux, as the highest power increase during the lactic phase may correspond to the peak increase in glycolytic activity. While the aerobic energy contribution only plays a minor role during a short maximal sprint, the phosphagen system and PCr degradation provide the majority of the energy required (Langley et al. 2024; Yang et al. 2023). However, PCr degradation appears to peak at approximately 1.3 s and declines thereafter (Maughan et al. 1997). Glycolysis is activated almost immediately at the beginning of the sprint and can possibly support or ensure the mobilization of power in this phase in addition to the already decreasing PCr degradation (Baker et al. 2010; Jones et al. 1985). Therefore, the superior predictive value of rel.maxΔ*P* can be attributed to its reduced sensitivity to alactic and aerobic energy contributions, making it a more precise indicator of glycolytic performance in maximal cycling sprints. This suggests its utility as a non-invasive metric for assessing glycolytic power and possibly in monitoring the effectiveness of training interventions, and identifying talent.

Interpreting the relationship between ΔBLC and relative power metrics requires careful consideration. Bundle and Weyand (2012) suggest that short sprints are “demand-driven” rather than “supply-limited,” meaning that glycolytic activity is a response to mobilized power rather than a determinant of performance. This is supported by the higher ΔBLC observed in high-power participants, likely reflecting differences in muscle fiber composition and neuromuscular coordination. Type II fibers, which dominate sprint-like activities (Andersen et al. 1994), are associated with faster contraction velocities, greater power output, and a higher reliance on glycolytic ATP resynthesis (Ricoy et al. 1998). These characteristics underlie their preferential recruitment during maximal sprints and their significant contribution to lactate formation. While the distribution of muscle fiber types is partially genetically determined, training can induce fiber type transitions (Simoneau and Bouchard 1995). Sprint, power, and strength training promote a shift toward fast-glycolytic fibers (Andersen et al. 1994; Liu et al. 2003), enhancing anaerobic energy production. Conversely, endurance training shifts fibers toward slow oxidative types, favoring aerobic energy pathways (Trappe et al. 2006). Consequently, sprinters, with a greater proportion of glycolytic fibers, rely heavily on anaerobic glycolysis during high-intensity efforts (Dunst et al. 2024). This adaptation is corroborated by evidence of increased glycolytic performance after a 6-week resistance training program, as demonstrated by improved outcomes in isokinetic strength tests (Nitzsche et al. 2020). The significantly greater rapid power increase during the lactic phase observed in the more powerful group is likely attributable to the preferential recruitment of type II fibers, resulting in elevated lactate production.

The results of this study could find practical application utilizing the *v*La_max_. The measurement of *v*La_max_ is used for example for metabolic profiling or to predict the performance in cycling (Hauser et al. 2014; Thron et al. 2024). The observed correlations between ΔBLC (or *v*La_max_) and sprint performance are in line with previous studies, which reported significant positive correlations for *P*_peak_ and *P*_mean_ across sports like cycling, running, rowing, and swimming, particularly for efforts lasting up to 1 min (Gupta et al. 2021, 2022; Korhonen et al. 2005; Lacour et al. 1990; Mavroudi et al. 2023; Ohkuwa et al. 1984; Quittmann et al. 2020, 2021; Weyand et al. 1994; Yang et al. 2023). The variability in the strength of correlations across studies may be influenced by factors such as test duration and the metrics analyzed, as longer test durations led to lower *v*La_max_ values or reduced lactate invasion into the blood compartment, but higher BLC_peak_ or ΔBLC values (Beneke et al. 2007; Langley et al. 2024).

However, there are still some concerns about the determination of *v*La_max_, particularly with regard to the alactic time (*t*_alac_). Only a poor to moderate reliability could be observed for methods to determine *t*_alac_ based on *P*_peak_ or a reduction of *P*_peak_ by 3.5%. The use of a fixed *t*_alac_ in this study may not reflect the individuality of athletes, but can improve the reliability and the ability to distinguish between different types of athletes (sprinter vs. endurance type) (Meixner et al. 2024). Yet there are conflicting results regarding the ability of a fixed *t*_alac_ to improve the reliability (Harnish et al. 2024). Taking into account the existing limitations, the recent findings could strengthen the use of *v*La_max_. The close relationship to the performance relative to the bodyweight during an isokinetic cycling sprint may help interpreting the importance of the *v*La_max_ of an athlete. This may be of further importance if future research can show adaptations of the power, like increases in rel.maxΔ*P* or *P*_mean_ accompanied by increases in *v*La_max_. To further consolidate the results of this study future research should also test if the observed correlations between ΔBLC and the rel.maxΔ*P* in the lactic phase are also evident in elite athletes.

### Limitations

The utilization of a lode ergometer in this study introduces methodological constraints that require careful interpretation. The ergometer’s design alters the temporal dynamics of the initial acceleration phase, compressing it and shifting peak power increases to the lactic phase. Consequently, the observed rapid rise in power during the lactic phase may only indirectly reflect maximal glycolytic flux under these conditions, potentially influencing the interpretation of power output profiles. To ensure comparability across studies using ergometers with power increases from the onset of exercise, a modified phase-separation approach is recommended. Specifically, the initial 2 s of power output should be attributed to neuromuscular factors, capturing rapid motor unit recruitment and the utilization of ATP and phosphocreatine reserves. Subsequent steep linear increases in power output likely represent high glycolytic energy flux and thus to the activation of anaerobic glycolysis.

Additionally, as this study employed a cycling sprint protocol, the findings cannot be generalized to other disciplines without further investigation. The specific nature of the exercise modality may limit the broader applicability of the results.

This study employed an isokinetic cycling sprint at a fixed pedaling frequency of 130 rpm. Previous research has demonstrated significant impacts of pedaling frequency on BLC_peak_, ΔBLC, *P*_peak_, and *P*_mean_ (Dunst et al. 2021; Haase et al. 2024; Jones et al. 1985). Consequently, the relationships between ΔBLC and performance metrics may vary when different pedaling frequencies are utilized. Future studies should examine whether these findings hold across different pedaling frequencies and exercise modalities to enhance the generalizability of the results.

The population in our study was heterogeneous with regard to the type of sport practiced and the neuromuscular and metabolic capacities of the participants. While correlations between BLC_peak_ or ΔBLC and performance (maximal and mean power) were also observed in highly trained athletes, it is possible that the observed correlation with the power increase in this study will not be seen or not as pronounced in a more homogeneous population (Hautier et al. 1994; Ohkuwa et al. 1984; Takei et al. 2018). In addition, the extent to which the results of this study apply to a female sample is unclear due to differences in glucose metabolism and differing lactate accumulation (Esbjörnsson-Liljedahl et al. 1999; Thron et al. 2024).

## Conclusions

These results suggest that absolute performance metrics may be confounded by neuromuscular factors, emphasizing the need to adjust for body weight (or fatigue free exercise-specific neuromuscular performance) to isolate metabolic performance more accurately. The distinct predictive potential of relative peak power increase and relative mean power likely reflects their sensitivity to different physiological processes. When normalized to body weight, *P*_mean_ provides a comprehensive measure of total energy supply, integrating contributions from alactic anaerobic, lactic anaerobic, and aerobic energy systems. However, this integrative metric may mask the contribution of individual energy systems. In contrast, the superior predictive value of the relative peak power increase observed during the lactic phase of exercise can be attributed to a large degree of independence from alactic and aerobic contributions, making it a more accurate indicator of glycolytic performance. As such, relative peak power increase during the lactic phase can serve as a valuable, non-invasive metric for evaluating glycolytic power and potentially monitoring training effectiveness, and identifying talent.

## Data Availability

The data is available on reasonable request from the corresponding author.

## Abbreviations

A: Extravascular increase in lactate concentration
ATP: Adenosine triphosphate
BLC: Blood lactate concentration
BLC_pre_: Capillary blood lactate concentration prior to the sprint test
BLC_peak_: Peak capillary blood lactate concentration following the sprint
BLC_max_: Maximal capillary blood lactate concentration following the sprint (determined by bi-exponential function)
ΔBLC: Blood lactate accumulation (BLC_max_–BLC_pre_)
ΔP: Power increase calculated by *P*_peak_/*t*_*P*__peak_
H +: Hydrogen ions
k_1_: Invasion constant of lactate into the blood compartment
k_2_: Elimination constant of lactate out of the blood compartment
PCr: Phosphocreatine
*P*_peak_: Peak power output
*P*_mean_: Mean power output
maxΔ*P*: Strongest power increase over 1 s
*r*: Pearson’s r correlation coefficient
*R*^2^: Coefficient of determination
rel*P*_peak_: *P*_peak_ relative to the body weight
rel*P*_mean_: *P*_mean_ relative to the bodyweight
rel.maxΔ*P*: MaxΔ*P* relative to the body weight
relΔ*P*: Δ*P* relative to the body weight
rpm: Revolutions per minute
SEE: Standard error of estimate
*t*_alac_: Fictional alactic time interval
TBLC_max_: Time to reach BLC_max_
*T*_load_: Duration of the sprint test (10 s)
*t*_*P*__peak_: Time to reach *P*_peak_
*v*La_max_: Maximal rate of blood lactate accumulation
